# Description of three new species of *Scolanthus* (Cnidaria, Anthozoa, Actiniaria, Edwardsiidae): first records of the genus from Japan

**DOI:** 10.3897/zookeys.794.25243

**Published:** 2018-11-01

**Authors:** Takato Izumi, Toshihiko Fujita

**Affiliations:** 1 Department of Biological Sciences, School of Science, The University of Tokyo, Bunkyo-ku, Tokyo 113-0033, Japan; 2 Department of Zoology, National Museum of Nature and Science, Tsukuba, Ibaraki 305-0005, Japan

**Keywords:** Aboral end, Ena Bay, nemathybome, Ogasawara Islands, sea anemone, edwardsiids, Sugashima Island, taxonomic key

## Abstract

*Scolanthus* is one genus of Edwardsiidae, a speciose family of burrowing worm-like sea anemones characterized by lacking a physa-like aboral end and by possessing nemathybomes on the whole body except at the distal end. This genus has been recorded worldwide, but there have been no specimens collected from Japan. In this study, we discovered four *Scolanthus* species in Japan for the first time: *Scolanthusarmatus* (Carlgren, 1931) and *Scolanthuskopepe***sp. n.** from the Ogasawara Islands, *Scolanthusena***sp. n.** from Ena Bay, Kanagawa, and *Scolanthusisei***sp. n.** from Sugashima Island, Mie.

## Introduction

The family Edwardsiidae is characterized by their worm-like bodies and is one of the major taxa in the order Actiniaria. Edwardsiidae contains approximately 90 species (Fautin 2013) and are diagnosed by having eight perfect mesenteries on the first cycle even as adults, while almost all other sea anemones have twelve perfect mesenteries in their first cycle (Carlgren 1949). The mesenterial arrangement of Edwardsiidae has been regarded for a long time as an ancestral character among actinarians as the arrangement is similar to those of several sea anemone larvae (reviewed in Daly 2002a, Uchida and Soyama 2001). However, this view has been challenged by the finding that the simplified mesenterial arrangement of this family may not be an ancestral character but is a secondary adaptation to infaunal life (e.g., Manuel 1981, Daly 2002b). Recently, this hypothesis was reinforced by a molecular phylogenetic study (Rodríguez et al. 2014).

In Japan, 13 species of five genera of the family Edwardsiidae have been reported (Table 1; Yanagi 2006, Sanamyan and Sanamyan 2013, Izumi et al. 2018). Of these, seven species are morphologically described in taxonomic papers (Carlgren 1921, 1931, 1940, Sanamyan and Sanamyan 2013, Stimpson 1856, Uchida 1932, Izumi et al. 2018) and the remaining five species have only been reported in field guide books without precise morphological information (Uchida 1965, Uchida and Soyama 2001).

The genus *Scolanthus* was established by Gosse (1853), who designated *Scolanthuscallimorphus* Gosse, 1853 as the type species by monotypy. However, *Scolanthus* was synonymized with *Edwardsia* by Gosse himself (Gosse 1855) and *S.callimorphus* was transferred to *Edwardsia*. Later, Manuel (1981) stated that this species did not belong to *Edwardsia*, and the genus *Scolanthus* was revived for the species and also came to include two species of the genus *Isoedwardsia* Carlgren, 1921. England (1987) agreed with this synonymization, and also transferred *Edwardsiaarmata* Carlgren, 1931 to *Scolanthus* (see also Daly 2002b, Daly and Ljubenkov 2008). Today, *Scolanthus* is distinguished from other genera by having nemathybomes and no physa in the aboral end (Manuel 1981, Daly and Ljubenkov 2008).

The study of Edwardsiidae in Japanese waters has not progressed much, and additionally the genus *Scolanthus* has not been reported from Japan thus far. In this research, we report *Scolanthusarmatus* (Carlgren, 1931) from the Ogasawara Islands, Japan, as well as discover and describe three new species from Japanese waters.

**Table 1.** All Edwardsiidae sea anemones recorded in Japanese waters. Note some researchers recently have advocated that *Metedwardsiaakkeshi* (and the genus *Metedwardsia*) do not belong to Edwardsiidae (Gusmão et al. 2016).

Species	Localities in Japan	Source
*Edwardsiajaponica* Carlgren, 1931	Misaki, Sagami Bay (Type locality)	Carlgren 1931
*Edwardsiaoctoradiata* Carlgren, 1931	Japan (Type locality)	Carlgren 1931
*Edwardsiaarctica* Carlgren, 1921	2300 m depth in Sea of Japan	Carlgren 1940
*Edwardsiasojabio* Sanamyan & Sanamyan, 2013	500–3500 m in Sea of Japan (Type locality)	Sanamyan and Sanamyan 2013
*Paraedwardsiacretata* (Stimpson, 1856)	Kagoshima Bay, Japan (Type locality)	Stimpson 1856
*Metedwardsiaakkeshi* (Uchida, 1932)	Akkeshi, Hokkaido (Type locality)	Uchida 1932
*Tempuractisrinkai* Izumi, Ise & Yanagi, 2018	Misaki, Sado, Toba (Type localities)	Izumi et al. 2018
**Species below were only included in field guidebook**		
*Edwardsiasipunculoides* (Stimpson, 1853)	Tohoku region	Uchida 1965
Edwardsianthuscf.pudica (Klunzinger, 1987)	Sagami Bay, Onagawa Bay (as *E.japonica* in Uchida, 1941 and 1965)	Uchida and Soyama 2001
*Edwardsianthusgilbertensis* (Carlgren, 1931)	Kabira Bay, Okinawa	Uchida and Soyama 2001
*Edwardsiahantuensis* England, 1987	Boso Pninsula, Chiba	Uchida and Soyama 2001
Edwardsiaaff.norvegica (Carlgren, 1942)	Kii Peninsula, Wakayama	Uchida and Soyama 2001

## Materials and methods

### Sample collection and preservation

**Figure 1.**
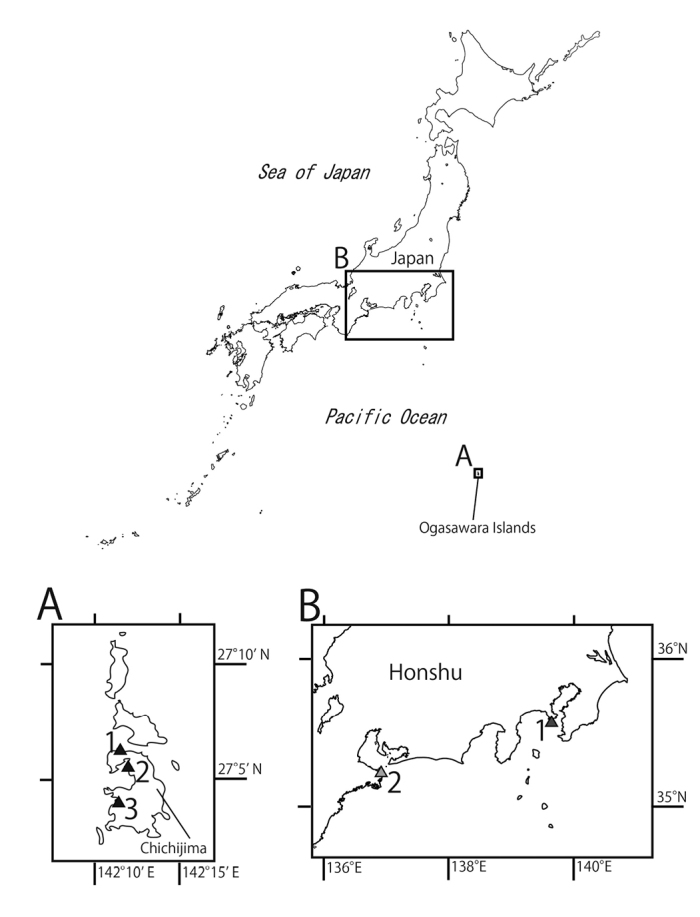
Sampling localities of *Scolanthus* specimens in this study. **A** Chichijima Island, Ogasawara Islands, Tokyo Metropolis **1** Miyanohama Coast **2** Seihyo Coast **3** Kopepe Coast **B** central Honshu **1** Ena Bay, Kanagawa Prefecture **2** Sugashima Island, Mie Prefecture.

The specimens were dug out from sandy seafloors using a shovel and a sieve, or from rocky substrates using a chisel, by wading, snorkeling, or scuba diving. Sampling was carried out around Chichijima Island in the Ogasawara Islands, in Ena Bay in Kanagawa, and around Sugashima Island in Mie, Japan (Fig. 1). All sea anemones were kept undisturbed in aquaria for several hours to several days after collection, until they were acclimated and completely spread and elongated their tentacles. Then, these relaxed specimens were anesthetized with magnesium chloride solution or l-menthol. For *Scolanthusena* sp. n. and the holotype specimen of *S.isei* sp. n., the whole body, except for a few tentacles, was fixed in 5% seawater formalin solution. Several tentacles were dissected from the body and preserved in 99.5% ethanol. As for *S.armatus* and *S.kopepe* sp. n., the tissue samples were cross-sectional sliced portions of the body instead of tentacles, which are too tiny to dissect exactly.

The examined specimens were deposited in the National Museum of Nature and Science, Tokyo (NSMT).

### Preparation of histological sections

Histological sections were made generally following standard protocols of Presnell and Schreibman (1997). The specimens of *Scolanthusarmatus*, *S.ena* and *S.isei* were dissected into different tissues (LIST), dehydrated by ethanol and xylene, embeded in paraffin, sliced into serial sections (8–10 µm thick) by using a microtome, mounted on glass slides, and stained with hematoxylin and eosin. Because *S.kopepe* sp. n. was too small to dissect, whole individuals were used to make sections by following the methods above.

### Cnidae observation

Cnidae were observed in the tentacle, actinopharynx, nemathybomes, column and filament; this genus does not have physa, so there are no cnidome data for the physa. Images of the cnidae were obtained by differential interference contrast microscopy (Yanagi et al. 2015). The length and width were measured using the software ImageJ v. 1.49 (Rasband 1997–2012). Cnidae nomenclature followed Mariscal (1974).

## Results

### Actiniaria Hertwig, 1882

#### Edwardsiidae Andres, 1881

##### Scolanthus

Taxon classificationAnimaliaActiniariaEdwardsiidae

Gosse, 1853

###### Diagnosis.

Edwardsiidae with body divisible into scapus and capitulum. Proximal part of body rounded, provided with nemathybomes and never forming physa. Nemathybomes scattered or forming longitudinal lines on scapus. At least eight microcnemes. Tentacle typically 16 to 20 in adults, arranged hexamerously, octomerously or decamerously. Tentacles on inner cycle shorter than those on outer cycle. Retractor muscles relatively large, well developed, and diffused, restricted or circumscribed. Parietal muscles distinct, symmetrical, well developed. Cnidome; spirocysts, basitrichs, microbasic *b*- and *p*-mastigophores (revised from Daly and Ljubenkov 2008).

###### Type species.

*Scolanthuscallimorphus* Gosse, 1853 by monotypy.

###### Origin of Japanese name.

New Japanese name: Ashinashi-mushimodoki-ginchaku-zoku. This new Japanese name is constructed from “ashi-nashi” (which means no-foot) and “mushimodoki-ginchaku” (which means worm-like, the Japanese name for edwardsiids). “Ashi-nashi” is named after the most characteristic feature of this genus; no differentiated physa from scapus.

###### Remarks.

The genus *Scolanthus* currently contains seven valid species (Fautin 2013) not including new species in the current study, and there also are two more species once accommodated within *Isoedwardsia*, which was synonymized to *Scolanthus* in Manuel (1981). This genus is widely distributed, from tropical (*S.armatus* [Carlgren, 1931]) to subarctic regions (*S.nidarosiensis* [Carlgren, 1942]). No *Scolanthus* species, however, have previously been collected from Japan. In this study, *Scolanthusarmatus* and three new species of *Scolanthus* are reported from Japan, representing the first records of this genus from Japan. Consequently, the number of *Scolanthus* species is now 12.

##### Scolanthus armatus

Taxon classificationAnimaliaActiniariaEdwardsiidae

(Carlgren, 1931)

Figs 2 , 6A , 7A–E

Edwardsia armata Carlgren, 1931: 2, figs 1–2; Carlgren 1949: 24
Scolanthus armatus : England 1987: 229, figs 13–14.

###### Material examined.

**Figure 2.**
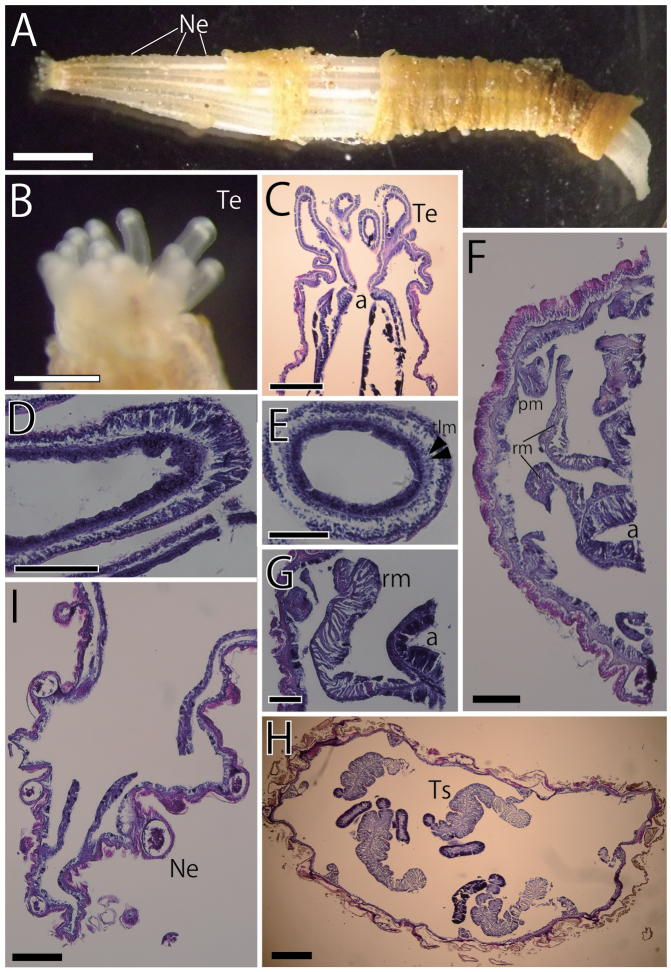
External and internal views of *Scolanthusarmatus* (NSMT-Co 1609). External views (**A, B**) and internal histological sections (**C–I**). **A** Whole living specimen of *S.armatus*. The periderm of aboral end was stripped off while the sampling **B** enlarged view of most distal part **C** longitudinal section of oral end **D** longitudinal section of a tentacle **E** transverse section of a tentacle **F** transverse section of actinopharynx of the longitudinal half of body **G** transverse section of mesentery **H** transverse section of filaments **I** longitudinal section of aboral end. Nemathybomes distributed up to the tip of aboral end. Abbreviations: a, actinopharynx; Ne, nemathybome; pm, parietal muscle; rm, retractor muscle; Te, tentacle; tlm, tentacular longitudinal muscle; Ts, testis. Scale bars: 5 mm (**A**); 1 mm (**B**); 500 µm (**C, F, H, I**); 100 µm (**D, E, G**).

NSMT-Co 1609, histological sections (7 slides), dissected tissues, tissues embedded in paraffin, and prepared nematocysts (6 slides), 27 June 2014, Seihyo Coast (Fig. 1A–2), Chichijima Island, Ogasawara Islands, Tokyo, Japan (27°09'47"N, 142°20'26"E), coral sand, 3 m depth, collected by scuba diving with a shovel and a sieve, by Takato Izumi.

###### Description.

*External anatomy.* Column rough, ca. 40 mm in whole length, and ca. 5 mm in width, worm-like form, and the proximal part narrower to some extent. The column consists of capitulum, scapulus and scapus. The most proximal part of column capitulum, distinct, extremely short, whitish and semitransparent, but scapulus and scapus indistinct. The periderm-like cuticle, brownish orange with no pattern in color, covering the whole column except capitulum and tentacle but easily stripped off from epidermis (Fig. 2A). The scapus beneath periderm semitransparent, with scattered small prominent nemathybomes but no papillae (Fig. 2A). Aboral end of the column tapered or flattened, not differentiated from scapus, with scattered nemathybomes (Fig. 2, A and 2I). No pedal disk, and no physa or physa-like structure. Tentacles in two cycles, 16 in number, eight in inner and eight in outer cycle (Fig. 6A). All tentacles long and slender, 1.5–2.0 mm in length, the inner tentacles as long as outer ones, transparent or semi-transparent, and capitated on their tip (Fig. 2B). Oral disk ca. 1.5 mm in diameter.

*Internal anatomy.* Eight perfect mesenteries, all macrocnemes. Four dorsal and ventral directives, and four lateral mesenteries not-paired with other macrocnemes, arranged in normal *Edwardsia* pattern (Fig. 6A). All macrocnemes present along whole length of the body from oral to aboral end, and bearing distinct retractor and parietal muscles. The retractor muscle of lateral mesenteries all ventrally facing (Fig. 6A). Eight tiny microcnemes, without muscles, confined only in distal-most part. Four microcnemes between dorsal directives and dorso-lateral mesenteries, two between dorso- and ventro-lateral mesenteries, and two between ventro-lateral mesenteries and ventral directives. Each tentacle between either exo- or endocoelic. (Fig. 6A). Retractor muscles strongly developed and diffused (Fig. 2F-H), pennon-like, configured with 15–30 muscular processes, each slightly branched (Fig. 2F). Parietal muscles with approximately 10 muscular processes (Fig. 2F). Actinopharynx short, ca. 2.5 mm in length, no distinct siphonoglyph (Fig. 2C). Tentacular circular muscle indistinct (Fig. 2D) and longitudinal muscle ectodermal and distinct (Fig. 2E). Mesoglea thin in the whole body, less than 100 µm even at the thickest part of body wall (Fig. 2C–I). Nemathybomes, ca. 100 µm in diameter, protrude from mesoglea on the column including the aboral end (Fig. 2I). Marginal sphincter muscle and basilar muscle absent (Fig. 2C, H). Gonads adjacent to the retractor muscle, relatively long (Fig. 2H). Testes between filament and retractor muscle, but no mature gametes in this specimen.

*Cnidome.* Basitrichs (in all tissues), spirocysts (in tentacles), microbasic *b*-mastigophores (in filaments) (Table 2, Fig. 7A–E). Basitrichs in filaments are distinguished into two types by their size.

###### Distribution.

Fiji (Carlgren 1931: type locality), Australia, Singapore (England 1987), and Japan (this study).

###### Origin of Japanese name.

New Japanese name: Ogasawara-ashinashi-mushimodoki. “Ogasawara” is the locality name. In Japanese waters, this species has been collected only in the Ogasawara Islands. “Ashinashi-mushimodoki” is short for the Japanese name of this genus (see the etymology of genus).

###### Remarks.

*Scolanthusarmatus* was originally described by Carlgren (1931) as *Edwardsiaarmata* when the genus *Scolanthus* was a junior synonym of *Edwardsia*. After *Scolanthus* was revived by Manuel (1981), this species was transferred to *Scolanthus* by England (1987). This species had no physa-like structure but many nemathybomes in the aboral end. This is the most characteristic feature of *Scolanthus*, and this feature is not found in *Edwardsia*. The specimen from the Ogasawara (Bonin) Islands almost completely agrees with the description of *Scolanthusarmatus* in England (1987); e.g. 16 tentacles which are capitated on the tip; rounded or flattened aboral end; scattered nemathybomes extending to the proximal end; strong and diffused retractor muscle (slightly branched muscular processes also correspond to England, 1987; fig. 13). The proportion and size of body is also similar to England’s description. There are, however, a few differences in the cnidome; England (1987) mentioned nothing about *b*-mastigophores in the description; stating that basitrichs and microbasic *b*-mastigophores could be distinguished by both the large diameter of the capsule and the broadened shaft shape (England 1991). However, both cnidomes are still easily confused and they are difficult to delineate. Thus, microbasic *b*-mastigophores with their broadened shape were judged by England (1987) to probably be basitrichs. This probability is reinforced by England (1987: table 6): two types of “basitrichs” in filament of *S.armatus*, but one type of basitrich with apparently broader capsules than the other type while the lengths are not different. In addition, there are very long basitrichs in our specimen, but they are few in number. If the numbers of these long basitrichs are very low, it is possible that England (1987) did not observe them. Moreover, the other cnidae size ranges resemble those of specimens of England (1987).

In conclusion, we identified this specimen as *S.armatus* because of its similarity in almost all morphological features to the original description of the species. The slight difference observed in the cnidome may be simply individual variation.

##### Scolanthus ena sp. n.

Taxon classificationAnimaliaActiniariaEdwardsiidae

http://zoobank.org/8EBE4524-8D01-48B0-A76C-BB0BBE423395

Figs 3 , 6B , 7F–I

###### Material examined.

*Holotype.*NSMT-Co 1610. One specimen cut into several parts, histological sections (23 slides) and prepared nematocysts (5 slides), 17 May, 2014, Ena Bay (Fig. 1B–1), Kanagawa, Japan, mud in the intertidal zone, collected by wading with a shovel, by Masanori Taru.

###### Description.

**Figure 3.**
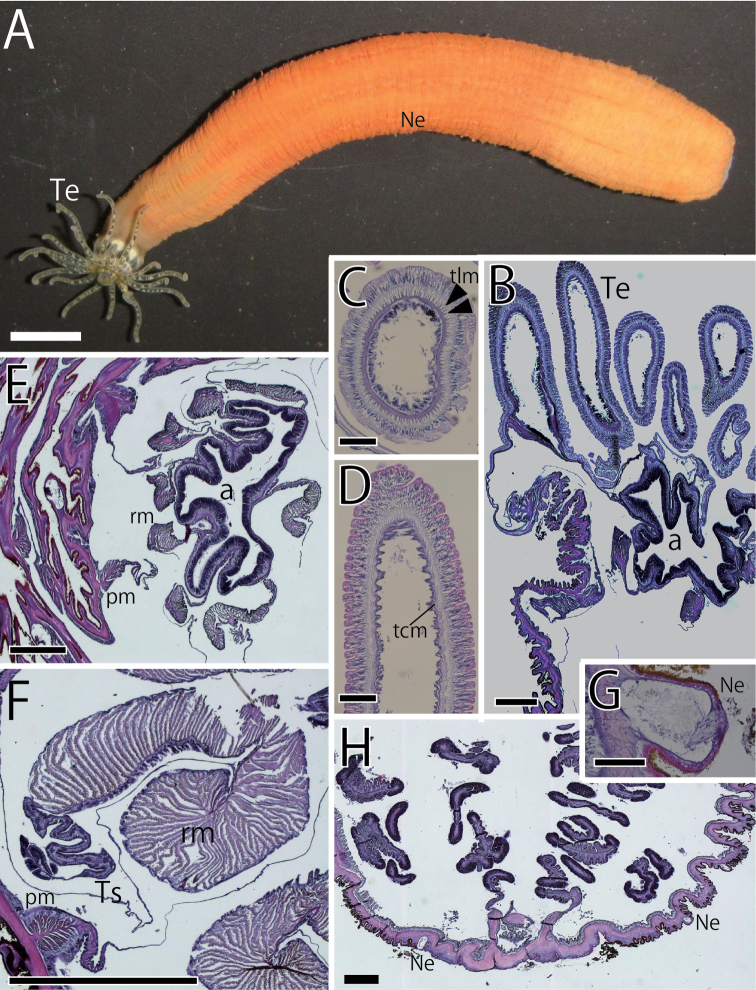
External and internal views of *Scolanthusena* sp. n. (NSMT-Co 1610)**. A** External view Whole living specimen of *S.ena***B–H** internal view **B** longitudinal section of distal end **C** transverse section of tentacle **D** longitudinal section of tentacle **E** transverse section of actinopharynx **F** enlarged view of mesentery **G** transverse section of nemathybome **H** longitudinal section of aboral end. Nemathybomes distributed up to aboral end. Abbreviations: a, actinopharynx; Ne, nemathybome; pm, parietal muscle; rm, retractor muscle; tcm, tentacular circular muscle; Te, tentacle; tlm, tentacular longitudinal muscle; Ts, testis. Scale bars: 5 mm (**A**); 1 mm (**B**); 500 µm (**E, F, H**); 100 µm (**C, D, G**).

*External anatomy.* Column rough, rugged and uneven, ca. 80 mm in whole length in holotype, and 10–15 mm in width, pipe-like in form both in living (Fig. 3A) and fixed specimen. The most upper part narrower to some extent. The most proximal part of column capitulum, dark brownish semitransparent, and the remaining part to aboral end scapus. The periderm of column orange, with no pattern in color, but thinner on the mesenteries so that the mesenterial line visible through the body wall. Scapus with scattered nemathybomes but no papillae. Aboral end rounded, not differentiated from scapus, with nemathybome (Fig. 3A, H). No pedal disk, but no physa or physa-like structure. Tentacles slender, no acrosphere, brownish, semi-transparent with white patch on each surface (Fig. 3A). Tentacles 20 in number, in two cycles; ten in inner and ten in outer cycle (Fig. 6B), 7.0–10.0 mm in length, longer than oral disk diameter and the inner tentacles shorter than outer ones. Oral disk ca. 5.0 mm in diameter. The mouth swelled and dome-like.

*Internal anatomy.* Eight perfect mesenteries, all macrocnemes. Four dorsal and ventral directives, and four lateral mesenteries not-paired with other macrocnemes (Figs 3E, 6B). All macrocnemes present along whole length of the body, from oral to aboral end and bearing distinct retractor and parietal muscles. The retractor muscle of lateral mesenteries all ventrally facing (Fig. 6B). Twelve tiny microcnemes, without muscles, confirmed only in distal-most part. Four microcnemes between dorsal directives and dorso-lateral mesenteries, four between dorso- and ventro-lateral mesenteries, and four between ventro-lateral mesenteries and ventral directives (Fig. 6B), an unusual arrangement for Edwardsiidae. Each tentacle between either exo- or endocoelic (Fig. 6B). Each retractor muscle pennon-like, restricted throughout the whole body (Fig. 3E, F), comparatively smaller next to actinopharynx but largely developed in lower part, limited in the part next to actinopharynx or filaments of each macrocneme (Fig. 3E, F). Muscle pennons consisting of approximately 30–60 muscular processes, some of which are well-branched into 10 or more branches (Fig. 3F). Parietal muscles with approximately 15 branched muscular processes (Fig. 3F). Actinopharynx short, no distinct siphonoglyph (Fig. 3E). Tentacular circular muscle endodermal (Fig. 3D) and longitudinal muscle ectodermal (Fig. 3C), both distinct. Mesoglea thickest in the aboral end, thick in body wall, approximately 70–120µm thick (Fig. 3B, F, H). However, mesoglea far thinner in actinopharynx and thinnest in mesenteries (Fig. 3E, F). Nemathybomes, around 200 µm in diameter, half buried into mesoglea on the column including the aboral end (Fig. 3G). Marginal sphincter muscle and basilar muscle absent (Fig. 3B, H). Gonads next the retractor muscle, comparatively long (Fig. 3F). Testes in gonads of holotype, between filament and retractor muscle.

*Cnidome.* Spirocysts (in tentacles), basitrichs (in every tissue), microbasic *b*-mastigophores (in filament) and microbasic *p*-mastigophores (in actinopharynx, and filament) (Table 2, Figs 7F–I). Basitrichs in actinopharynx and nemathybome are distinguished into two types by their size. No nematocysts in body wall.

###### Distribution.

Ena Bay, Kanagawa. Known only from the type locality.

###### Etymology.

The species epithet is named after the type locality, Ena Bay. The word “ena” is a noun in apposition. **Origin of Japanese name**: new Japanese name: taru-ashinashi-mushimodoki. “Taru” is the name of the collector of this new species.

###### Remarks.

**Figure 4.**
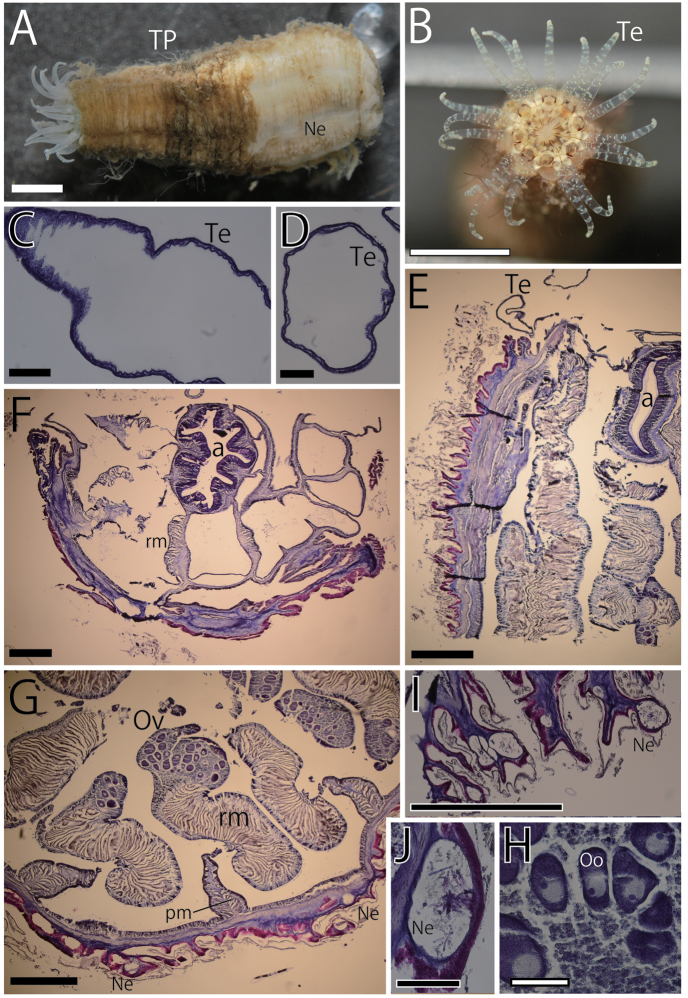
External and internal views of *Scolanthusisei* sp. n. (NSMT-Co 1611)**. A, B** External view **A** whole preserved specimen of *S.isei***B** view of tentacular circle of living specimen **C–J** internal view (histological section) **C** longitudinal section of tentacle **D** transverse section of tentacle **E** longitudinal section of oral end **F** transverse section of actinopharynx (half of body) **G** enlarged view of mesentery **H** enlarged view of gonad. Several matured oocytes contained **I** densely distributed nemathybomes **J** enlarged view of transverse section of nemathybome. Abbreviations: a, actinopharynx; Ne, nemathybome; Oo, oocyte; Ov, ovary; pm, parietal muscle; rm, retractor muscle; Te, tentacle; TP, trichome-like structure on periderm. Scale bars: 5 mm (**A, B**); 500 µm (**E, F, G, I**); 100 µm(**C, D, H, J**).

*Scolanthusena* sp. n. has 20 tentacles, as do *Scolanthusignotus* (Carlgren, 1920) and *S.isei* sp. n.: other edwardsiid species have 16 tentacles (England 1987, Daly and Ljubenkov 2008, and this study). Small and large types of basitrichs in nemathybomes of *S.ena* are far smaller and far larger, respectively, than basitrichs in nemathybomes of *S.ignotus* (Carlgren 1920, England 1987). Moreover, *S.ena* sp. n. is 80 mm in body length, approximately three to four times longer than the 20–30 mm of *S.ignotus*. *Scolanthusena* sp. n. is different from *S.isei* sp. n. in its tentacular arrangement (Fig. 6B, C), structure of column surface (periderm of *S.ena* sp. n. does not have trichome-like structures [Fig. 4A] unlike *S.isei* sp. n.; the nemathybomes of *S.ena* sp. n. are far more sparse than those of *S.isei* sp. n.), body size (*S.ena* sp. nov. is far bigger than *S.isei* sp. n.), and cnidome (only *S.ena* sp. n. has microbasic *b*-mastigophores in their filaments) (see Table 2). In addition, *S.isei* sp. n. lives in cavities of bare rocks, a different habitat compared to that of *S.ena* sp. n. (see Remarks of *S.isei*).

This species is one of the biggest species in the genus *Scolanthus*: all previously reported species of nominal *Scolanthus* have bodies less than 80 mm in length (Gosse 1853, McMurrich 1893, Carlgren 1920, Carlgren 1921, Pax 1924, Carlgren 1931, Carlgren 1942, Daly and Ljubenkov 2008).

Despite several sample collection surveys at Ena Bay, *S.ena* sp. n. was collected only once, and no specimens have been collected from any other locality. It is said by local people that the environment of Ena Bay has changed from several decades ago; the bottom of bay was previously rocky, and a muddy flat has formed in recent years by inflow of sediment. Considering some *Scolanthus* live in rocky habitats compared to other edwardsiids (e.g. *S.isei* sp. n.), the primary habitat of *S.ena* sp. n. might be rocky, and perhaps the numbers of individuals have decreased in Ena Bay by recent rapid changes in the environment. It is difficult, however, to examine this hypothesis because Edwardsiidae sea anemones living in or between rocks often cannot be collected easily even if there are many individuals present.

Even though there is only one specimen of *S.ena*, the character differences from other *Scolanthus* species make it obvious that this specimen is not a formerly described *Scolanthus* species. Examination of additional specimens in the future may help better delineate this species.

##### Scolanthus isei sp. n.

Taxon classificationAnimaliaActiniariaEdwardsiidae

http://zoobank.org/AF324E73-96BD-4AB9-BA19-13744DFA2E6A

Figs 4 , 6C , 7J–M

###### Material examined.

*Holotype.*NSMT-Co 1611. One specimen cut into several parts, histological sections (5 slides) and prepared nematocysts (5 slides), on August 1, 2014, Sugashima Island (Fig. 1B–2), Mie, Japan (34°29'4"N, 136°52'31"E), cavity of a rock at a depth around 50 cm at low tide, collected by snorkeling by hand, by Yuji Ise. *Paratype*. NSMT-Co 1612. Histological sections (5 slides), damaged slightly when collected, on August 4, 2014, Sugashima, Mie, Japan (34°28'51"N, 136°52'46"E), cavity of a rock at a depth around 30 cm at low tide, collected by hand, by Yuji Ise.

###### Description.

*External anatomy.* Column rough, rugged and uneven, ca. 30 mm in whole length in fixed holotype, and 10–12 mm in width, truncated cone-like form both in living and fixed (Fig. 4A) specimen, comparatively tubby form for edwardsiids. Paratype a little small, ca. 18 mm in length and ca. 9 mm in width. Upper part narrower than lower part. No apparent capitulum, all parts of column uniformly scapus. Periderm brownish or whitish, no pattern in color, with trichome-like structure (Fig. 4A), and easily stripped. Column with highly densely scattered nemathybome but no papillae, and the surface on the mesenteries slightly sunken (Fig. 4A). Aboral end rounded, not differentiated from scapus, with nemathybome. No pedal disk, but no physa or physa-like structure (Fig. 4A, G). Tentacles slender, no acrosphere, completely transparent and white patches or stripes on each surface. Tentacles 20 in number, in two cycles; eight in inner and twelve in outer cycle (Figs 4B, 6C), 5.0–7.0 mm in length, longer than oral disk diameter and the inner tentacles shorter than outer ones. Oral disk ca. 4.0 mm in diameter. Mouth not swollen.

*Internal anatomy.* Eight perfect mesenteries, all macrocnemes. Four dorsal and ventral directives, and four lateral mesenteries not paired with other macrocnemes (Fig. 6C). All macrocnemes present along whole length of the body, from oral to aboral end, and bearing distinct retractor and parietal muscles. The retractor muscle of lateral mesenteries all ventrally facing (Fig. 6C). Twelve tiny microcnemes, without muscles, confined only in distal-most part. Four microcnemes between dorsal directives and dorso-lateral mesenteries, four between dorso- and ventro-lateral mesenteries, and four between ventro-lateral mesenteries and ventral directives, in unusual arrangement for Edwardsiidae. One tentacle each between exocoels and endocoels (Fig. 6C). Retractor muscles pennon-like, diffused throughout the whole body, smaller next to the actinopharynx (Fig. 4F), but largely developed and almost integrated to gonads in lower part (Fig. 4G). Each muscle pennons consisting of approximately 20–30 single or slightly branched muscular processes (Fig. 4F, G). Parietal muscles with approximately 15–20 muscular processes (Fig. 4G). Actinopharynx short, no distinct siphonoglyphs (Fig. 4F). Both tentacular circular muscle and longitudinal muscle too weakly developed to observe (Fig. 4C, D). Mesoglea thickest in the body wall and aboral end, approximately 200–300 and in some parts over 500 µm thick. However, mesoglea far thinner in actinopharynx and mesenteries (Fig. 4E, F, G), and thinnest in tentacles (Fig. 4C, D). Nemathybomes, around 150 µm in diameter, protruded from body wall (Fig. 4I) in the column but a little buried into the mesoglea in the aboral end (Fig. 4G, J). Marginal sphincter muscle and basilar muscle absent (Fig. 4E, G). Gonads next to retractor muscle, short, and wide (Fig. 4G). Ovary between retractor muscle and filament, and oocytes in gonads of holotype.

*Cnidome.* Spirocysts (in tentacles), basitrichs (in all tissues), microbasic *b*-mastigophores (in actinopharynx) and microbasic *p*-mastigophores (in filament) (Table 2, Fig. 7J–M). Basitrichs in tentacles, nemathybomes and filaments are distinguished into two types by their size. No nematocysts in body wall.

###### Distribution.

Southwest coast of Sugashima Island, Mie. Known only from the type locality.

###### Etymology.

The species name was named after Yuji Ise, the collector of both holotype and paratype specimens. **Origin of Japanese name**: New Japanese name: sugashima-gareba-ashinashi-mushimodoki; “gareba” means rocky seashore, the habitat where this species inhabits.

###### Remarks.

In terms of having 20 tentacles, *Scolanthusisei* sp. n. resembles *S.ignotus* and *S.ena* sp. n., while all other edwardsiid species have 16 tentacles (England 1987, Daly and Ljubenkov 2008, and this study). This species is different from *S.ignotus* in having two types of basitrichs of different sizes in nemathybomes (Carlgren 1920), and the larger type is far bigger than the basitrichs of *S.ignotus*. The differences between *S.isei* sp. n. and *S.ena* sp. n. are principally regarding the tentacular arrangement and body size (see Remarks of *S.ena* sp. n.).

Exceptionally for edwardsiids, this species inhabits in the cavities of the underside of boulders, adhering by their aboral end, on rocky seashores. Edwardsiids usually inhabit in sand or mud, and only two species had been reported from other different environments: *Edwardsiellaandrillae* Daly, 2013 living on ice (Daly et al. 2013), and *Tempuractisrinkai* Izumi, Ise & Yanagi, 2018 living in sponges (Izumi et al. 2018).

##### Scolanthus kopepe sp. n.

Taxon classificationAnimaliaActiniariaEdwardsiidae

http://zoobank.org/45D2F38B-EB2B-46FF-8B41-CC570EE35A15

Figs 5 , 6A , 7N–R

###### Material examined.

*Holotype.*NSMT-Co 1613, histological sections (5 slides), and tissue for DNA analysis, 26 June 2014, Kopepe Seashore (Fig. 1A–3), Chichijima Island, Ogasawara Islands, Tokyo, Japan (27°3'52"N, 142°11'33"E), coral sand at 1 m depth, collected by snorkeling with a shovel and a sieve by Takato Izumi. *Paratypes.*NSMT-Co 1614, dissected tissues, and prepared nematocysts (5 slides), at same date, place, by same method, and collector as NSMT-Co 1613.; NSMT-Co 1615, prepared nematocysts (5 slides), at same date, place, by same method, and collector as NSMT-Co 1613.; NSMT-Co 1616, histological sections (2 slides), and dissected tissues, at same date, place, by same method, and collector as NSMT-Co 1613 ; NSMT-Co 1617, whole specimen, at same date, place, by same method, and collector as NSMT-Co 1613; NSMT-Co 1618, histological sections (5 slides) 21 June 2014, Miyanohama coast (Fig. 1A–1), Chichijima Island, Ogasawara Islands, Tokyo, Japan (27°6'18"N, 142°11'39"E), coral sand at 7 m depth, collected by scuba diving with a shovel and a sieve by Takato Izumi.

###### Description.

**Figure 5.**
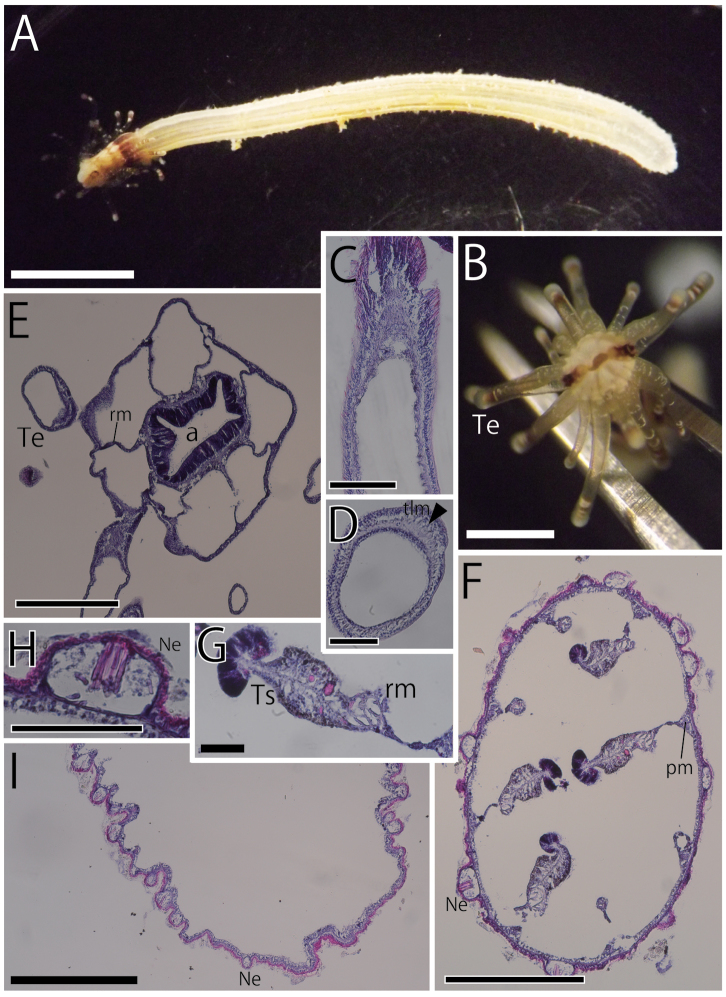
External and internal views of *Scolanthuskopepe* sp. n. **A, B, F–I**NSMT-Co 1613 **C–E**NSMT-Co 1616 **A, B** external view **A** whole living specimen of *S.kopepe***B** enlarged view of tentacular circle **C–I** internal view (histological section) **C** longitudinal section of tentacle **D** transverse section of tentacle **E** transverse section of actinopharynx **F** transverse section of filaments **G** enlarged view of mesentery **H** transverse section of nemathybome **I** longitudinal section of aboral end. Nemathybomes distributed up to the tip of aboral end. Abbreviations: a, actinopharynx; Ne, nemathybome; pm, parietal muscle; rm, retractor muscle; Te, tentacle; tlm, tentacular longitudinal muscle; Ts, testis. Scale bars: 5 mm (**A**); 1 mm (**B**); 500 µm (**E, F, G, I**); 100 µm (**C, D, H, J**).

**Figure 6.**
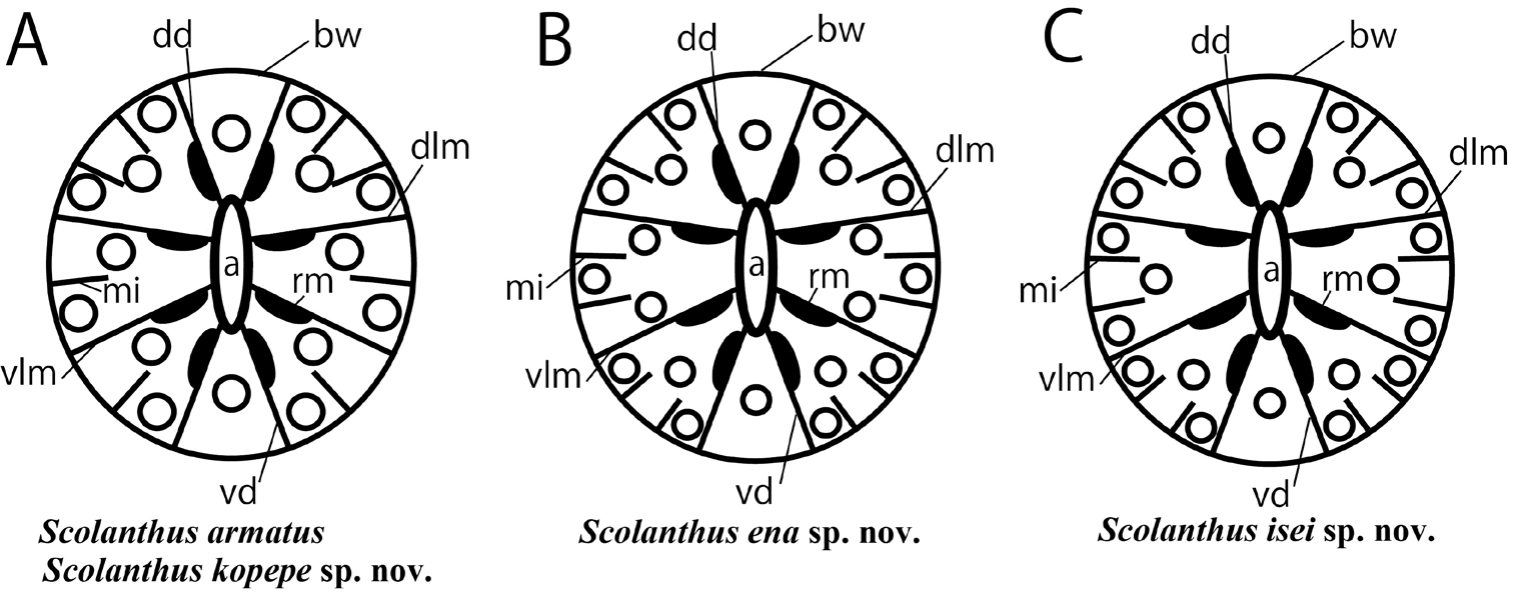
Schematic representation of tentacular and mesenterial arrangements. **A***Scolanthusarmatus* and *S.kopepe* sp. n. **B***S.ena* sp. n. **C***S.isei* sp. n. Abbreviations: a, actinopharynx; bw: body wall; dd, dorsal directive; dlm, dorso-lateral mesentery; mi, microcneme; rm, retractor muscle; vd, ventral directive; vlm, ventro-lateral directive. White circles indicate the locus of tentacles.

*External anatomy.* Column comparatively smooth, ca. 15–25 mm in whole length (25.0 mm in holotype), and 1–2 mm in width (1.8 mm in holotype), naked and extremely long and narrow pipe-like form both in living (Fig. 5A) or fixed specimens. The upper part as narrow as lower part. The most proximal part capitulum, transparent and thin. The remaining part to aboral end of body scapus, with white to pale yellow periderm, and with scattered nemathybome but no papillae. Aboral end a little rounded or tapered, not differentiated from scapus, with nemathybomes (Fig. 5A, I). Tentacles slender, capitated on the tentacle tip, transparent with white patch on each tentacle tip, 1.5–2.0 mm in length, longer than oral disk diameter, but well expanded and contacted. Tentacles 16 in number, in two cycles; eight in inner and eight in outer cycle, same as *Edwardsia*’s arrangement (Fig. 6A), the inner tentacles shorter than outer ones. Oral disk ca. 1 mm in diameter, white with a brownish red stripe from ventral side to dorsal side. The mouth not swollen.

*Internal anatomy.* Eight perfect mesenteries, all macrocnemes. Four dorsal and ventral directives, and four lateral mesenteries not-paired with other macrocnemes, arranged in normal *Edwardsia* pattern (Figs 5E, 6A). All macrocnemes present along whole length of the body, from oral to aboral end, and bearing retractor and parietal muscles. The retractor muscle of lateral mesenteries all ventrally facing (Fig. 6A). Eight tiny microcnemes, without muscles, only in distal most part. Four microcnemes between dorsal directives and dorso-lateral mesenteries, two between dorso- and ventro-lateral mesenteries, and two between ventro-lateral mesenteries and ventral directives, common arrangement in Edwardsiidae. Each tentacle between either exo- or endocoelic. (Fig. 6A). Each retractor muscles pennon-like, diffused, small and weak next to actinopharynx (Fig. 5E) but restricted, comparatively well developed, and limited besides gonads and integrated into them in lower part (Fig. 5F, G). Muscle pennons consisting of approximately 2–5 simple muscular processes (Fig. 4G). Parietal muscles of macrocnemes not very distinct (Fig. 5G). Actinopharynx very short, no distinct siphonoglyph (Fig. 5E). Tentacular circular muscle indistinct (Fig. 5C) and longitudinal muscle ectodermal and distinct (Fig. 5D). Marginal sphincter muscle and basilar muscle absent. Mesoglea generally thin in the whole body, a few micrometers even in body wall (Fig. 5E–G, I). Nemathybomes, approximately 100 µm in diameter, protrude from the body wall as their diameter far larger than the thickness of mesoglea. Gonads next the retractor muscle, but no mature gametes in specimens we observed (Fig. 5G).

**Figure 7.**
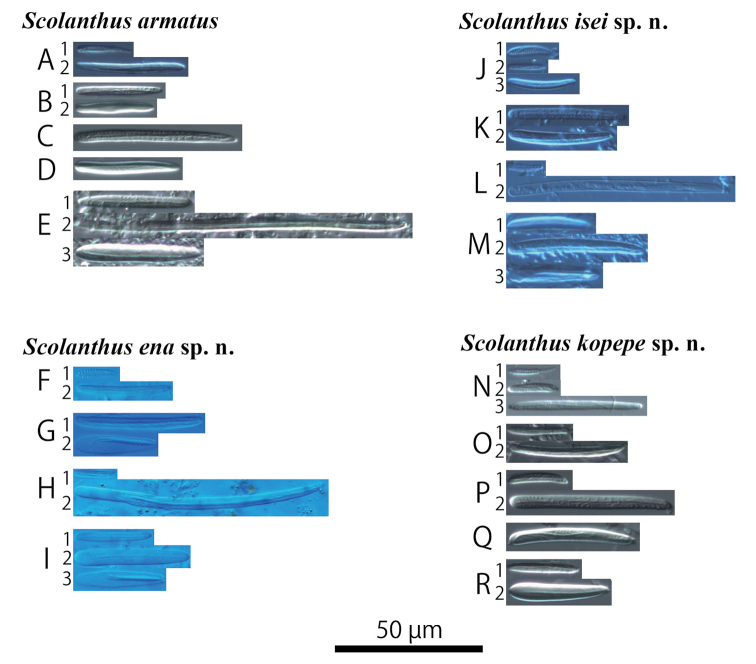
Cnidae of *Scolanthus* species. **A–E***S.armatus* (NSMT-Co 1609) **A1** spirocyst in tentacle **A2** basitrich in tentacle **B1** basitrich in actinopharynx **B2** microbasic *b*- mastigophore in actinopharynx **C** basitrich in nemathybome **D** basitrich in column **E1** small basitrich in filament **E2** large basitrich in filament **E3** microbasic *b*- mastigophore in filament **F–I***S.ena* sp. n. (NSMT-Co 1610) **F1** spirocyst in tentacle **F2** basitrich in tentacle **G1** basitrich in actinopharynx **G2** microbasic *p*- mastigophore in actinopharynx **H1** small basitrich in nemathybome **H2** large basitrich in nemathybome **I1** basitrich in filament **I2** microbasic *b*- mastigophore in filament **I3** microbasic *p*- mastigophore in filament **J–M***S.isei* sp. n. (NSMT-Co 1611) **J1** spirocyst in tentacle **J2** small basitrich in tentacle **J3** large basitrich in tentacle **K1** basitrich in actinopharynx **K2** microbasic *b*- mastigophore in actinopharynx **L1** small basitrich in nemathybome **L2** large basitrich in regular tentacles **M1** small basitrich in filament **M2** large basitrich in filament **M3** microbasic *p*- mastigophore in filament **N–R***S.kopepe* sp. n. (NSMT-Co 1614) **N1** spirocyst in tentacle **N2** small basitrich in tentacle **N3** large basitrich in tentacle **O1** small basitrich in actinopharynx **O2** large basitrich in actinopharynx **P1** small basitrich in nemathybome **P2** small basitrich in nemathybome **Q** basitrich in column **R1** basitrich in filament **R2** microbasic *b*- mastigophore in filament.

*Cnidome.* Spirocysts (in tentacles), basitrichs (in all tissues), microbasic *b*-mastigophores (in actinopharynx, column and filament) (Table 2, Fig. 7N-R; holotype). Basitrichs in tentacle, column and nemathybomes are distinguished into two types by their size.

###### Distribution.

Chichijima Island, Ogasawara. Known only from the type locality.

###### Etymology.

Most of the specimens were collected from Kopepe Coast. “Kopepe” is the name of the native people that used to live in the Ogasawara Islands, who had emigrated from the Gilbert Islands, now the Republic of Kiribati. The word “kopepe” is a noun in apposition. **Origin of Japanese name**: New Japanese name: kopepe-ashinashi-mushimodoki. “Kopepe” is originated from same coast.

###### Remarks.

This species resembles not only *Scolanthusarmatus* but also *S.scamiti* Daly & Ljubenkov, 2008, *S.triangulus* Daly & Ljubenkov, 2008, *S.curacaoensis* (Pax, 1924), *S.nidarosiensis* (Carlgren, 1942) and *S.callimorphus* Gosse, 1853 in terms of having 16 tentacles (Gosse 1853, Manuel 1981, England 1987, Daly and Ljubenkov 2008). *Scolanthuskopepe* sp. n. is similar to *S.armatus*, and both are found around the same island. *Scolanthuskopepe* sp. n., however, is morphologically distinguishable by several points as below: *S.kopepe* sp. n. is smaller than *S.armatus*, one-third to a half in length and one-fifth to one-third in width (the specimen of *S.armatus* is even far bigger than living *S.kopepe* sp. n.); *S.kopepe* sp. n. has brownish red stripe from ventral side to dorsal side on oral disk and white patch on capitated tentacle tip, both features are not present on *S.armatus*; the number of muscular processes of both retractor muscles and parietal muscles of *S.kopepe* sp. n. are far fewer than those of *S.armatus* (Figs 2G, H, 5E, G; England 1987); moreover, the *S.kopepe* sp. n. holotype has two types of basitrichs in the tentacles, actinopharynx and nemathybomes while *S.armatus* has only one type (Table 2). The body of *S.kopepe* sp. n. is slender and uniform in width (both in living and preserved specimens) while that of *S.scamiti* is stout and increasing in width toward to the aboral end (Daly and Ljubenkov 2008). Besides, the basitrichs in the nemathybomes of *S.kopepe* sp. n. are two types while those of *S.scamiti* are only one type (Table 2). *Scolanthuskopepe* sp. n. is 15–25 mm in body length while *S.triangulus* has a maximum body length of 11 mm (Daly and Ljubenkov 2008), smaller than *S.kopepe* sp. n. Furthermore, basitrichs of *S.triangulus* are over 63 µm (Daly and Ljubenkov 2008; table 3), larger than both types of basitrichs of *S.kopepe* sp. n. Basitrichs in the nemathybomes of *S.callimorphus* are of only one type and are over 60 µm in length (Manuel 1981, p 265), while *S.kopepe* sp. n. has two types of basitrichs in the nemathybomes and both of them are less than 60 µm in length. *Scolanthuscuracaoensis* has far larger body, 45 mm in length (Pax 1924), and has a well-developed, circumscribed retractor muscle and rounded distinct parietal muscle (Pax 1924; Figs 4, 5) while *S.kopepe* sp. n. has diffused and undeveloped retractor and indistinct parietal muscle. *Scolanthusnidarosiensis* lives in the deep sea of a cold region (125–150 m depth of Norway; Carlgren 1942) in contrast to *S.kopepe* sp. n., which lives in shallow waters in the subtropical region. The retractor muscles of *S.nidarosiensis* are well developed and muscle processes were obviously branching (Carlgren 1942; fig. 71) while those of *S.kopepe* sp. n. are far less developed with simple processes. Moreover, nemathybomes of *S.nidarosiensis* contain longer basitrichs than the large basitrichs of *S.kopepe* sp. n., and *S.nidarosiensis* has only one type of basitrich (Carlgren 1942) while *S.kopepe* sp. n. has two types of basitrichs.

*Scolanthuskopepe* sp. n. usually lives in coral sand, and prefers dark environments under large rocks.

**Table 2.** Cnidae of four Japanese *Scolanthus* species. –absent. n = number of observed cnidae. **A**–**R** indicate figures of each kind of cnidae in Figure 7.

	Fig	* Scolanthus armatus *				Fig	*S.ena* sp. n.				Fig	*S.isei* sp. n.				Fig	*S.kopepe* sp. n.			
(specimen)		NSMT–Co 1609					NSMT–Co 1610					NSMT–Co 1611					NSMT–Co 1614			
		Length × Width (µm)			n		Length × Width (µm)			n		Length × Width (µm)			n		Length × Width (µm)			n
**Tentacle**																				
Spirocysts	A	11.79–20.69 × 1.74–3.70	17.02 × 2.56	2.11 × 0.37	45	F	11.15–20.90 × 2.11–3.21	15.57 × 2.62	2.00 × 0.27	53	J	10.99–16.03 × 2.35–3.76	13.52 × 3.13	1.19 × 0.35	40	O	10.40–18.29 × 1.55–2.80	14.99 × 2.08	2.08 × 0.29	47
Basitrichs		13.05–54.36 × 2.70–5.25	32.65 × 3.58	13.69 × 0.47	71		21.19–38.34 × 2.37–3.51	31.38 × 2.97	2.88 × 0.25	62		small 10.05–12.97 × 1.82–2.77	11.58 × 2.33	0.70 × 0.31	22		small 11.29–17.27 × 2.80–4.10	14.69 × 3.39	1.66 × 0.35	35
												large 19.10–26.60 × 3.04–4.52	22.54 × 3.71	1.64 × 0.33	59		large 31.91–55.17 × 2.70–4.92	45.82 × 3.97	5.67 × 0.46	73
**Actinopharynx**																				
Basitrichs	B	19.44–39.57 × 2.67–4.75	29.10 × 3.57	4.30 × 0.51	40	G	small 22.55–26.92 × 2.39–2.77	24.74 × 2.58	5.27 × 0.70	2	K	21.91–39.78 × 3.06–4.65	32.01 × 3.93	4.79 × 0.42	55	P	small 14.94–28.33 × 2.32–3.77	20.27 × 3.13	1.80 × 0.35	22
							large 37.46–51.19 × 2.95–4.62	43.98 × 3.90	3.12 × 0.32	65							large 33.75–46.69 × 3.14–4.61	39.83 × 3.88	2.97 × 0.33	38
Microbasic *b*–mastigophores		22.17–34.53 × 3.48–5.19	28.32 × 4.09	2.81 × 0.40	42		–	–	–	–		25.14–38.09 × 4.24–6.43	31.02 × 5.45	2.85 × 0.41	49		–	–	–	–
Microbasic *p*–mastigophores		–	–	–	–		21.67–30.55 × 4.94–6.93	27.16 × 5.68	1.69 × 0.50	22		–	–	–	–		–	–	–	–
**Nemathybome**																				
Basitrichs	C	36.20–62.95 × 3.41–5.80	52.02 × 4.69	7.15 × 0.56	54	H	small 9.74–15.15 × 2.20–3.05	12.07 × 2.53	1.38 × 0.23	16	L	small 9.68–12.35 × 1.96–3.23	11.02 × 2.40	1.34 × 0.36	5	Q	small 18.89–24.97 × 2.67–4.33	21.30 × 3.61	1.72 × 0.35	21
							large 63.93–105.59 × 3.41–5.40	83.26 × 4.26	7.85 × 0.39	47		large 62.84–84.98 × 3.76–6.16	73.91 × 4.87	9.76 × 0.60	52		large 29.79–58.01 × 2.80–5.20	44.81 × 4.45	7.41 × 0.49	56
**Column**																				
Basitrichs	D	10.47–49.97 × 2.76–5.00	18.16 × 3.56	10.93 × 0.59	21	–	–	–	–	–		–	–	–	–	R	14.28–50.47 × 3.90–4.15	39.57 × 4.69	8.26 × 0.55	31
**Filament**																				
Basitrichs	E	small 31.29–43.01 × 3.48–5.43	37.17 × 4.37	2.90 × 0.36	70	I	33.37–45.48 × 3.72–5.56	38.12 × 4.63	2.78 × 0.48	34	M	small 18.18–26.59 × 2.28–3.61	21.68 × 3.02	2.32 × 0.36	21	S	19.57–29.72 × 2.70–4.25	23.32 × 3.60	2.11 × 0.31	66
		large 95.62–114.46 × 2.67–4.16	102.12 × 3.64	8.28 × 0.69	3							large 38.66–49.82 × 4.56–6.65	44.45 × 5.60	2.50 × 0.52	41					
Microbasic *b*–mastigophores		33.80–45.69 × 5.21–7.90	43.19 × 6.54	2.14 × 0.71	21		34.26–39.98 × 5.11–6.55	36.85 × 5.80	1.91 × 0.46	12		–	–	–	–		32.30–39.62 × 5.24–7.77	35.88 × 6.22	1.68 × 0.51	57
Microbasic *p*–mastigophores		–	–	–	–		23.49–29.63 × 5.69–7.73	27.44 × 6.30	1.80 × 0.58	9		31.88–33.97 × 5.20–6.12	33.03 × 5.63	0.70 × 0.36	5		–	–	–	–

## Supplementary Material

XML Treatment for Scolanthus

XML Treatment for Scolanthus armatus

XML Treatment for Scolanthus ena

XML Treatment for Scolanthus isei

XML Treatment for Scolanthus kopepe
